# Neuroleptic malignant syndrome with thyroid disorder: An unusual case report

**DOI:** 10.1097/MD.0000000000008191

**Published:** 2017-09-29

**Authors:** Fan Zhang, Parisa Kanzali, Vadim Rubin, Chris Paras, Joel Goldman

**Affiliations:** a Department of Internal Medicine, Brookdale University Hospital and Medical Center, Brooklyn; b Ross University School of Medicine, Portsmouth, Dominica; c Division of Endocrinology, Brookdale University Hospital and Medical Center, Brooklyn, New York.

**Keywords:** chlorpromazine, hypothyroidism, neuroleptic malignant syndrome (NMS)

## Abstract

**Rationale:**

Neuroleptic malignant syndrome (NMS) is a life threatening neurologic emergency associated with neuroleptic or antipsychotic agent use. NMS is rarely related to thyroid disease.

**Patient concerns:**

We report a case of NMS in a 66-year-old male with past medical history of paranoid schizophrenia on chlorpromazine, diabetes, hypertension and asthma, who presented with a humeral fracture after a fall. Patient developed hyperpyrexia, altered consciousness, autonomic instability, elevated serum creatine kinase (CK) without rigidity.

**Diagnoses:**

CT head and workup for infection were negative. Electroencephalogram (EEG) showed generalized slow wave activity. Ultrasound revealed a large goiter with nodules.

**Interventions:**

Chlorpromazine was stopped due to concern of NMS. Patient was treated with cooling, fluid and electrolyte maintenance.

**Outcomes:**

Patient slowly improved and CK level normalized. Thyroid-stimulating hormone (TSH) level trended down from 10.2 mIU/L to 0.02 mIU/L. Patient was discharged with aripiprazole.

**Lessons:**

Hypothyroidism predisposes patients to NMS by altering central dopaminergic systems. The typical symptoms may be masked by hypothyroidism. Thyroid dysfunction should be excluded in all patients with NMS. Discontinuing antipsychotic agents decreases TSH levels which maybe due to the negative feedback of dopaminergic activity. This is the first case report describing dramatic changes in TSH after discontinuing chlorpromazine in NMS.

## 1 Introduction

Neuroleptic malignant syndrome (NMS) is the disturbance of hypothalamic and basal ganglia dopaminergic function. It may occur during treatment with neuroleptic or antipsychotic drugs.^[1]^ The tetrad of syndrome are mental status changes, rigidity, fever, and dysautonomia. The typical symptoms may be masked by hypothyroidism which also predisposes the patient to NMS by a general increase in brain dopaminergic activity.^[2,3]^ We report the first case with dramatic changes in thyroid-stimulating hormone (TSH) after discontinuing chlorpromazine in a patient of NMS with schizophrenia. Thyroid dysfunction should be excluded in a patient with NMS.

## 2 Case report

Patient is a 66-year-old African America male with past medical history of paranoid schizophrenia, diabetes, hypertension, and asthma. The patient felt dizzy and fell down on his right shoulder. He missed his morning insulin dose. Patient was alert, awake, oriented, and complained of right arm pain. On physical examination, patient had ecchymosis over the right upper arm, BP 90/68 mmHg, heart rate (HR) 66, and respiratory rate (RR) 18. Laboratory investigation revealed glucose 455 mg/dL, bicarbonate 19 mEq/L, venous blood gas (VBG) pH 7.27, creatinine 1.3, white blood cell (WBC) 7.3, and TSH 10.2 mIU/L (normal range 0.465–4.68 mIU/L). Patient was treated with intravenous hydration, subcutaneous insulin, and chlorpromazine 200 mg 3 times daily, which he had taken for 10 years previously for schizophrenia. Computed tomography (CT) of the head showed mild cortical atrophy with no acute changes. X-ray revealed mildly displaced fracture of the right humeral neck. Orthopedic surgery suggested no surgical intervention. On the second day, glucose level was 123, VBG pH 7.44, and creatinine 0.4 (normal). However, patient developed tachycardia with HR 110 and BP 144/90 mmHg. Over the next 3 days, patient's BP fluctuated from 103/70 to 150/94 mmHg and HR from 109 to 128. Patient was lethargic with slurring, mumbling, and mostly inaudible speech. He was able to follow simple commands and name 3 objects. On day 6, patient developed a fever of 38.4 °C (101.1 °F), tachycardia HR 130, BP 151/94 mmHg which was significantly increased from BP 113/75 mmHg within the past 24 hours. On day 7, patient was stuporous with fever 40.1 °C (104.2 °F), BP 119/67, HR 142, and tachypnea RR 29. CT head was negative for acute changes. EEG revealed generalized slowing. CT chest angiogram ruled out pulmonary embolic disease. Screening for infection was negative. Lumbar puncture was attempted without success. Patient had no nuchal rigidity or stiffness. Chlorpromazine was discontinued on the night of day 7 due to the concern of NMS. Patient was treated with vigorous cooling, fluid, and electrolyte maintenance.

**Figure 1:**
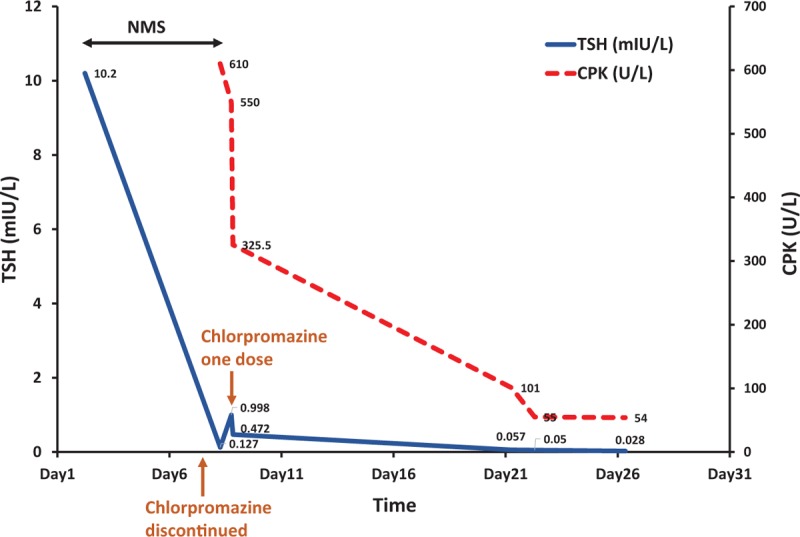
Discontinuing chlorpromazine resolved NMS and decreased TSH level. After 1 dose of chlorpromazine 200 mg (arrow), the TSH level rose initially and then trended down. NMS = neuroleptic malignant syndrome, TSH = thyroid-stimulating hormone.

On the morning of day 8, patient was lethargic with improved vital signs: T 37 °C (98.6 °F), BP 104/69 mmHg, HR 125. Notably, TSH decreased to 0.127 mIU/L with free T4 1.77 ng/dL (normal range 0.78–2.19 ng/dL) with elevated CK 610 U/L (normal range 55–170 U/L), and WBC 10.4. For an unclear reason, patient received 1 dose of chlorpromazine 200 mg in the morning. Repeated labs showed an increase in TSH 0.998 mIU/L with the same level of free T4 1.77 ng/dL. At night, the TSH dropped to 0.472 mIU/L with free T4 1.73 ng/dL and CK decreased to 550 U/L (Fig. 1). CT chest, abdomen, and pelvis showed no acute pulmonary or intra-abdominal findings, but revealed diffusely enlarged and heterogeneous thyroid gland.

Chlorpromazine was discontinued again and serial CK levels showed a declining trend from 610 to 384, 137, 119, 101, 72 U/L within the next 12 days (Fig. 1). His temperature varied from 36.4 °C (97.5 °F) to 37.5 °C (99.5 °F). Patient was still lethargic and had recurrent tachycardia with HR ranging from 94 to 158. Transthoracic echocardiogram revealed vigorous systolic function of left ventricle without regional wall motion abnormalities or structural heart disease. Holter monitor showed sinus tachycardia with average HR range between 101 and 142 per minute with occasional premature ventricular contractions and premature atrial contractions. On day 13, patient showed negative symptoms of alogia and flat affect with staring into space with delayed and minimal responses. He seemed internally preoccupied. His speech was slurred and difficult to understand. Patient stated he was hearing voices telling him to take his medications.

**Figure 2:**
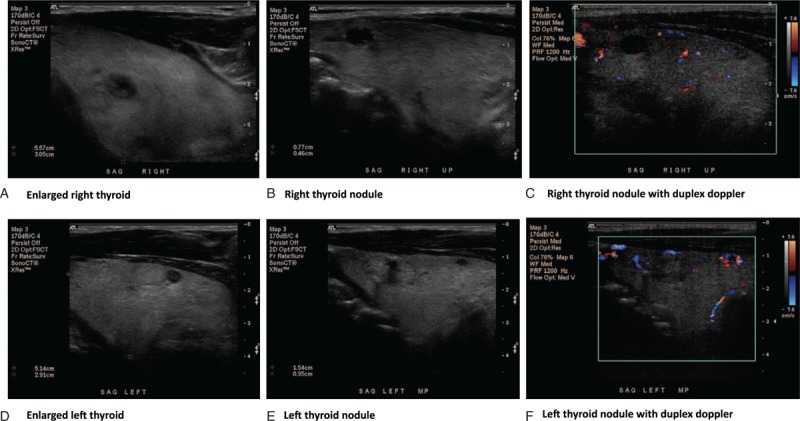
Diffuse enlarged thyroid gland with multiple nodules was shown in thyroid sonogram.

On day 21, patient had an episode of agitation. Patient was started on aripiprazole 5 mg daily per psychiatric consultation. His vital signs were within normal range. TSH was 0.05 mIU/L and free T4 was 2.14 ng/dL. Thyroid sonogram showed prominent enlarged thyroid gland with right thyroid lobe 7.4 cm × 1.9 cm × 3.4 cm and left thyroid lobe 6.6 cm × 2.4 cm × 3.0 cm. There are bilateral thyroid nodules with 2 nodules in the right thyroid lobe and 3 nodules on the left side. The largest nodule was 1.9 cm × 1.1 cm × 1.4 cm (Fig. 2).

Patient's delusion and agitation slowly remitted and was stabilized on aripiprazole. He was discharged on day 27 with T 36.7 °C (98 °F), BP 120/70 mmHg, HR 94, and CK level 54 U/L. TSH was low 0.028 mIU/L and free T4 was normal 1.81 ng/dL. Thyroid stimulating immunoglobulin, thyroid peroxidase antibodies, and thyroglobulin antibody were negative. Patient's movement and mental status returned to his baseline and he was able to answer questions and ambulate. Patient's past medical record was obtained showing that he was diagnosed with schizophrenia since the age of 24 years and was hearing voices on and off during his life. He was euthyroid one and a half years previously with TSH 1.21 mIU/L. He was discharged to be followed by psychiatry and endocrinology.

## 3 Discussion

NMS is a life-threatening neurological emergency associated with the use of neuroleptic agents which block dopamine transmission and characterized by a tetrad clinical syndrome of mental status change, fever, rigidity, and autonomic instability.^[4]^ The incident rates of NMS range from 0.02% to 3% among patients taking neuroleptic agents^[4,5]^ and the mortality is estimated between 10% and 20%.^[6]^ NMS is seen in every class of neuroleptic drugs with the most often occurring in the typical high potency neuroleptic agents (e.g., haloperidol, fluphenazine).^[7]^ Low potency (e.g., chlorpromazine), atypical antipsychotic drugs (e.g., clozapine, risperidone, olanzapine), and antiemetic drugs (e.g., metoclopramide, promethazine) have been implicated as well.^[8,9]^

There are several risk factors for development of NMS, such as dehydration,^[8,10]^ polypharmacy, depot neuroleptic, metabolic encephalopathy, and acute medical illness (e.g., trauma, surgery, and infection).^[4,6,11]^ In our patient, a fracture after a fall along with hyperglycemia associated with acidosis might have precipitated his development of NMS in the background of hypothyroidism with TSH of 10.2 mIU/L.

The typical clinic presentations of NMS usually evolves over 1 to 3 days with mental status changes as the initial symptoms, followed by rigidity, then hyperthermia and autonomic dysfunction. Atypical NMS occurs in milder cases, diagnosed early cases or associated with lower potency agents.^[7]^ The symptom of rigidity or fever may be mild, delayed, and even absent.^[5,8,12]^ The diagnosis of NMS should be considered when any 2 of the tetrad of symptoms are present in the setting of neuroleptic agents use. The degree of CK elevation is correlated with the severity of rigidity and prognosis of NMS. Although CK is typically more than 1000 IU/L and can be as high as 100,000 IU/L, normal CK can be seen if no clear rigidity developed.^[4,5,10,13]^ Our patient exhibited NMS symptoms without rigidity which maybe due to the milder form of NMS with the use of the low potency antipsychotic agent, chlorpromazine. On the other hand, his TSH was 10.2 mIU/L on admission compared with 1.2 mIU/L one and a half year earlier. This suggests that new onset of hypothyroidism may predispose the patient to NMS along with other precipitating factors including metabolic dysfunction and trauma. Furthermore, hypo-metabolic state with flaccidity of hypothyroidism may mask rigidity.

Hypothyroidism is often not diagnosed. Several cases of hypothyroidism with NMS have been reported.^[2,3,14–16]^ (Table 1) Hypothyroidism was found in both the initial episode of NMS and the recurrence.^[2]^ In the background of thyroid hormone deficiency, antipsychotic medications can even precipitate myxedema coma (MC).^[3,17]^ Interestingly, in 1 case of MC, similar to our patient, CK normalized after discontinuing antipsychotics for 12 days and patient was also discharged on hospital day 27 with aripiprazole.

**Table 1:**
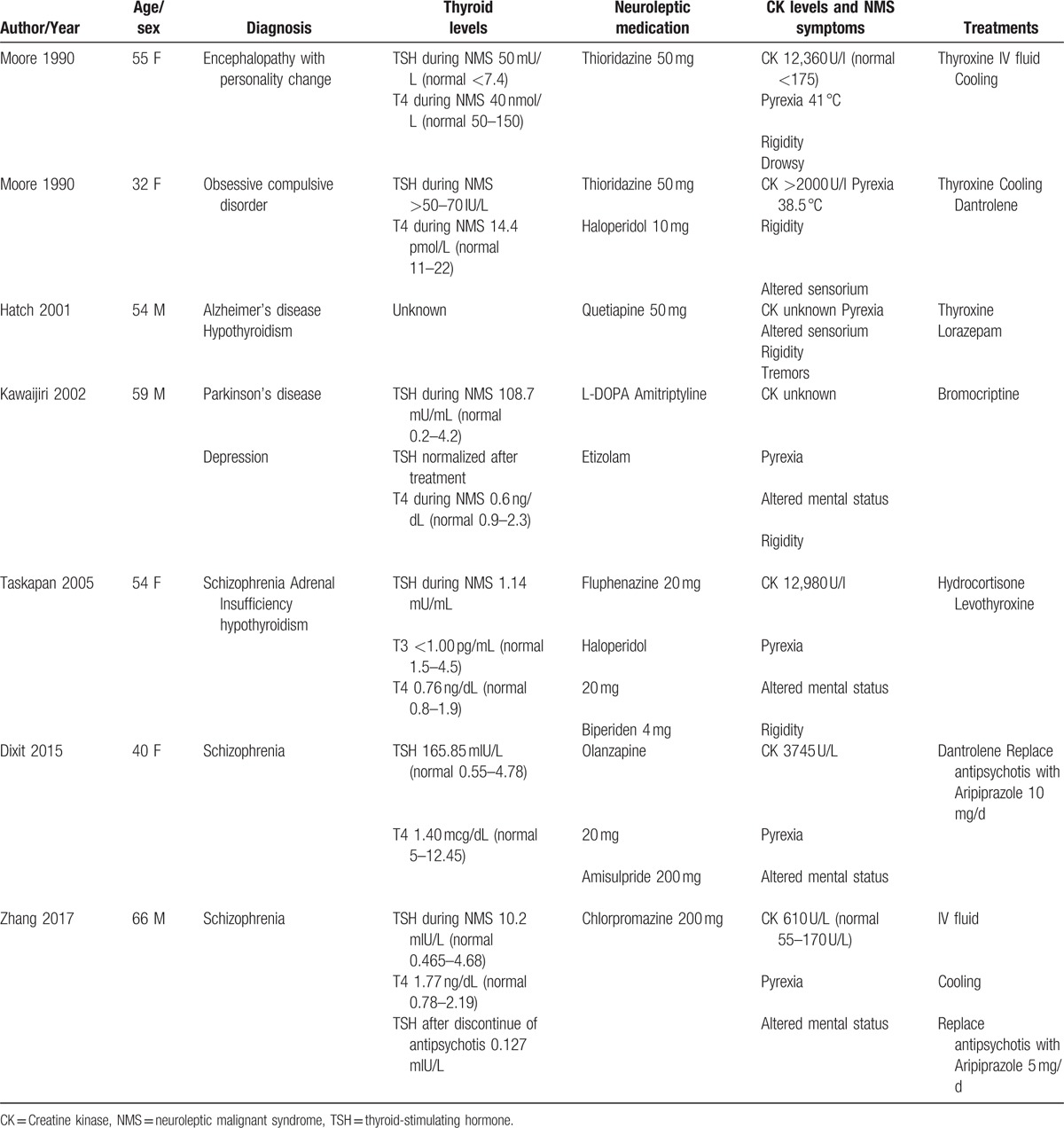
Case reports of NMS and hypothyroidism.

The relationship between chlorpromazine and thyroid function has been studied in the past at the thyroid gland level, circulation level, and hypothalamic-pituitary-thyroid (HPT) axis level. It may interfere with iodine capture by thyroid cells, increase deiodination, and exert a thyroid immunogenic effect.^[18]^ At the circulation level, drug-iodine complexes have formation constants, Kc, that estimate the electron-donating potential of the drug. Phenothiazines with high Kc (e.g., chlorpromazine) may lead to iatrogenic hypothyroidism through increased protein-bound iodine.^[19]^ At the thyroid gland level, chlorpromazine treatment has been shown to result in lowered serum T4 levels and decreased radioactive uptake of iodine by the thyroid gland in animal studies.^[20,21]^ At the HPT axis level, chlorpromazine can cause changes in TSH levels by inducing formation of antithyroid antibodies.^[18,22]^ Our patient, however, tested negative for thyroid stimulating immunoglobulin, thyroid peroxidase antibodies, and thyroglobulin antibodies. Infrequently, phenothiazines may induce thyroiditis and a hyperthyroid state. This is unlikely to have played a role in our patient's autonomic instability because the free T4 level was within normal limits and thyroid sonogram revealed chronic changes with multiple nodules in a bilateral enlarged thyroid.

The diagnostic criteria for NMS was established by an international multispecialty consensus group based on positive clinical and laboratory findings as well as the exclusion of alternative causes.^[23,24]^ Our patient scored 73 based on these criterias, including exposure to a dopamine antagonist within past 72 hours (score 20), hyperthermia (>38 °C or >100.4 °F on at least 2 occasions) (score 18), mental status alteration (score 13), sympathetic nervous system lability with BP elevation (>25% above baseline) and BP fluctuation (>20 mmHg diastolic changes or >25 mmHg systolic change within 24 hours) (score 10), hypermetabolism (HR >25% above baseline), RR increase (>50% above baseline) (score 5), and negative workup for infectious, toxic, metabolic, or neurologic causes (score 7). The initial level of CK during the early onset of NMS was unknown in this patient, which could be more than at least 4 times the upper limit of normal 170 U/L (score 10) based on the trending down pattern of CK from 610 to 550 U/L on the same day and then 384, 166, 137 U/L, on the next several days, respectively. The single most important treatment in NMS is removal of the causative agent. The mean recovery times of NMS are 7 to 11 days.^[8,10]^ To minimize risk of recurrence, guidelines of how to restart neuroleptic medications were recommended.^[4]^

**Figure 3:**
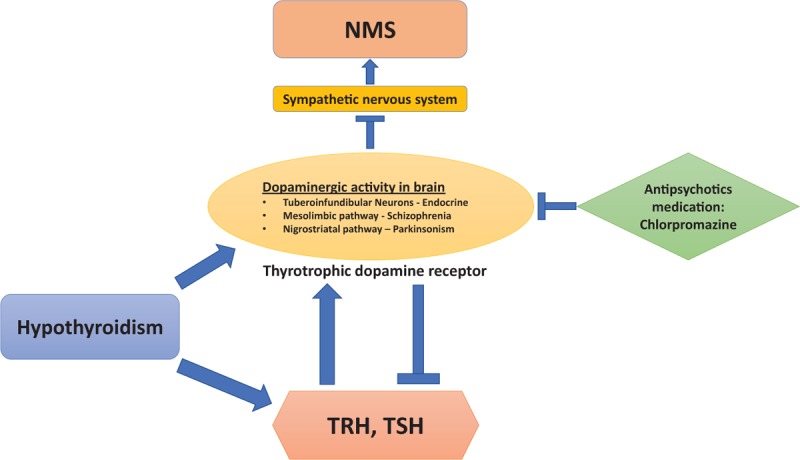
Pathogenesis of NMS in hypothyroidisms: dopaminergic activity is regulated by TSH, TRH, and antipsychotic medications. NMS = neuroleptic malignant syndrome, TRH = thyrotropin-releasing hormone, TSH = thyroid-stimulating hormone,

The pathogenesis of NMS is related to dopamine receptor blockade by antipsychotic medications (Fig. 3). Its effect in the hypothalamus causes hyperthermia and dysautonomia.^[25,26]^ The interference with nigrostriatal dopamine pathways leads to Parkinsonian type symptoms, including rigidity and tremor^[26,27]^; the effect on tuberoinfundibular dopamine pathway results in hyperprolactinemia and changes of anterior pituitary hormones; the disruption of the sympathetic nervous system leads to increased muscle tone and metabolism, ineffective heat dissipation, labile blood pressure, and HR.^[28]^ The direct toxic effect by neuroleptics on skeletal muscle also contribute to the rigidity and muscle damage.^[27,28]^

The general increase in dopaminergic activity secondary to hypothyroidism in the central dopamine systems predisposes the patient to NMS.^[2]^ Thyrotropin-releasing hormone induced increases of the sensitivity of postsynaptic dopamine receptors in the striatum and limbic forebrain lead to compensatory changes in motor control systems.^[29]^ Therefore, NMS can develop when the excess dopaminergic action is inhibited by neuroleptic agents.^[2]^ It has been confirmed in a hypothyroid animal model that dopaminergic activity is increased in the tuberoinfundibular neurons and elevated TSH levels cause increases of thyrotrophic dopamine receptor numbers.^[30]^

On the other hand, dopaminergic activity inhibits TSH release as a negative feedback. When chlorpromazine was discontinued in our patient, the increased dopaminergic activity could inhibit TSH release. This is suggested by the trending down of TSH from 10.2 to 0.988, 0.472, 0.05, and then 0.028 mIU/L (Fig. 1). Interestingly, when one dose of chlorpromazine was given back after it was initially discontinued on hospital day 3, TSH increased from 0.127 to 0.998 mIU/L. This further supports that dopaminergic activity inhibits TSH secretion. We are the first to report such TSH changes after discontinuing chlorpromazine in NMS.

## 4 Conclusion

We report a rare case of NMS with thyroid dysfunction. Hypothyroidism predisposes patients to NMS by altering central dopaminergic tracts which are vulnerable to neuroleptic blockade by anti-psychotic agents. Physicians should be alert to recognize the atypical manifestations of NMS in hypothyroidism patients. Neuroleptic agents should be stopped with suspicion of NMS. Thyroid dysfunction should be excluded in patients of NMS.
